# Correction

**DOI:** 10.1080/20002297.2024.2350309

**Published:** 2024-05-15

**Article title**: The effects of nitrate on the oral microbiome: a systematic review investigating prebiotic potential

**Authors**: Siobhan P. Moran, Bob T. Rosier, Fiona L. Henriquez and Mia C. Burleigh

**Journal**: *Journal of Oral Microbiology*

**Bibliometrics**: Volume 16, Number 01, pages 1-14

**DOI**: https://doi.org/10.1080/20002297.2024.2322228

It has been noted by the authors that disclosure statement was not included in the published article. This has been placed below. This correction has not changed the description, interpretation, or the original conclusions of the article. The authors apologize for any inconvenience caused.

